# Multifactorial Optimization of Contrast-Enhanced Nanofocus Computed Tomography for Quantitative Analysis of Neo-Tissue Formation in Tissue Engineering Constructs

**DOI:** 10.1371/journal.pone.0130227

**Published:** 2015-06-15

**Authors:** Maarten Sonnaert, Greet Kerckhofs, Ioannis Papantoniou, Sandra Van Vlierberghe, Veerle Boterberg, Peter Dubruel, Frank P. Luyten, Jan Schrooten, Liesbet Geris

**Affiliations:** 1 Prometheus, Division of Skeletal Tissue Engineering, KU Leuven, Leuven, Belgium; 2 Department of Materials Engineering, KU Leuven, Heverlee, Belgium; 3 Department of Development and Regeneration, Skeletal Biology and Engineering Research Center, KU Leuven, Leuven, Belgium; 4 Biomechanics Research Unit, Université de Liege, Liège, Belgium; 5 Polymer Chemistry and Biomaterials Group, University of Ghent, Ghent, Belgium; 6 Department of Mechanical Engineering, Biomechanics Section, KU Leuven, Heverlee, Belgium; Pennsylvania State Hershey College of Medicine, UNITED STATES

## Abstract

To progress the fields of tissue engineering (TE) and regenerative medicine, development of quantitative methods for non-invasive three dimensional characterization of engineered constructs (i.e. cells/tissue combined with scaffolds) becomes essential. In this study, we have defined the most optimal staining conditions for contrast-enhanced nanofocus computed tomography for three dimensional visualization and quantitative analysis of in vitro engineered neo-tissue (i.e. extracellular matrix containing cells) in perfusion bioreactor-developed Ti6Al4V constructs. A fractional factorial ‘design of experiments’ approach was used to elucidate the influence of the staining time and concentration of two contrast agents (Hexabrix and phosphotungstic acid) and the neo-tissue volume on the image contrast and dataset quality. Additionally, the neo-tissue shrinkage that was induced by phosphotungstic acid staining was quantified to determine the operating window within which this contrast agent can be accurately applied. For Hexabrix the staining concentration was the main parameter influencing image contrast and dataset quality. Using phosphotungstic acid the staining concentration had a significant influence on the image contrast while both staining concentration and neo-tissue volume had an influence on the dataset quality. The use of high concentrations of phosphotungstic acid did however introduce significant shrinkage of the neo-tissue indicating that, despite sub-optimal image contrast, low concentrations of this staining agent should be used to enable quantitative analysis. To conclude, design of experiments allowed us to define the most optimal staining conditions for contrast-enhanced nanofocus computed tomography to be used as a routine screening tool of neo-tissue formation in Ti6Al4V constructs, transforming it into a robust three dimensional quality control methodology.

## Introduction

The field of tissue engineering (TE) is evolving towards the development of complex, three-dimensional (3D) constructs (i.e. cells/tissue combined with scaffold) to mediate the repair of severe defects. In order to facilitate the successful clinical implementation of these constructs an in-depth understanding of how they develop as well as the optimization of the developed procedures and the availability of robust 3D quality assessment tools becomes essential.

Currently, the standard technique for evaluating tissue formation is histological sectioning. It has a high discriminative power, both on tissue and cellular level. However, it shows limited potential for quantifying 3D tissue formation as it is destructive and costly in terms of time and resources. Most importantly, in standard settings it only allows assessment of tissue distribution in 2D, with loss of information due to a restricted sectioning orientation and with limited depth resolution [1–3]. Other standard techniques to assess the quality of a construct are Live/Dead, DNA content (providing cell number estimation), histology and weight measurements. Although Live/Dead staining gives additional important information concerning cell state, it has limitations for internal, 3D visualization of tissue formation in a construct. Both DNA content and weight measurements are bulk measurement techniques not providing spatial information. Techniques such as confocal or multiphoton microscopy offer a potential for 3D visualization of cells and tissues. However limited depth resolution (~300μm) and limited options for detailed quantification hinders their performance when clinically relevant sized or opaque samples are to be analyzed. Ultrasound and magnetic resonance imaging could obtain significantly higher imaging depths, but limitations in the spatial resolution result in significant restrictions for quantitative 3D analysis. Therefore, there is a need for more advanced, quantitative 3D imaging techniques [4].

Recent advances in 3D imaging techniques and image analysis have demonstrated the potential of the currently applied methods and their limitations for accurate analysis. In particular X-ray micro and nanofocus computed tomography (micro and nanoCT) have been frequently applied as 3D quantitative imaging techniques to assess mineralized skeletal tissues [5, 6]. However, because of their low X-ray attenuation, soft tissue contrast is inherently poor in absorption mode imaging. Phase contrast imaging could be a solution [7, 8]. Due to the electromagnetic properties of the X-rays, a phase shift of the waves can be induced as a result of differences in the refractive index of different materials while passing through an object. Taking this phase shift into account, the contrast sensitivity will be increased, which is especially for low absorbing materials an important benefit. As this technology requires sophisticated X-ray optics and preferably monochromatic X-rays, it is mostly available using synchrotron radiation [9], although recently also desktop CT devices allowing phase contrast imaging have become available [10, 11].

A recent shift in micro- and nanoCT imaging focuses on the use of X-ray opaque contrast agents for visualizing soft tissues, such as cartilage [12–15], blood vessels [16–18] and connective tissues [19, 20]. Specifically in the case of neo-tissues (cells and extracellular matrix), we have shown proof-of-concept of contrast-enhanced nanoCT (CE-nanoCT) for the 3D visualization and quantification of neo-tissue within constructs formed in bioreactor cultures [21]. Two tissue-specific contrast agents were used to stain the neo-tissue, i.e. Hexabrix and phosphotungstic acid (PTA). As opposed to the standard techniques mentioned earlier, it was shown that CE-nanoCT could allow 3D qualitative and quantitative structural and spatial assessment of the *in vitro* engineered neo-tissue, created during static and bioreactor cell culturing in titanium alloy scaffolds, revealing 3D neo-tissue distribution.

Although this study indicated the potential of CE-nanoCT, the staining conditions, which were based on preliminary internal experiments [14, 15], resulted in datasets with sub-optimal image quality. This was due to limited contrast in combination with scaffold-induced imaging artifacts, such as beam hardening and streaks. Although extensive image processing enabled quantitative interpretation of the datasets, no automated image analysis was possible yet; this posed considerable limitations to the proposed methodology. A recent publication that assessed several contrast agents for CE-CT indicated the necessity to optimize the staining parameters for obtaining quantitative datasets. It was demonstrated that for the same staining conditions each contrast agent displayed agent-specific contrast enhancement levels and penetration depth, while the sample size further affected its performance [22].

An additional concern for the use of contrast agents is their potential to influence the integrity of the stained tissue. Iodine potassium iodide (I2KI) was shown to cause substantial soft tissue shrinkage dependent on the concentration, staining time and tissue structure/composition [23]. Also PTA was shown to introduce shrinkage of brain and muscle tissue, although in a lesser extent than I2KI [24]. For some studies, the compositional information of the soft tissues in the sample, only obtainable by the staining, was most important [25–27], although structural tissue alterations due to dehydration or staining were noticed [25]. However, when aiming at using the CE-CT data for morphometric and volumetric analyses, the degree of specimen shrinkage, and potential deformation, is an important consideration.

In the current study, we applied a ‘Design of Experiments’ (DoE) approach, a statistical method for planning experiments, for a multiparametric investigation of the influence of staining parameters (staining time and concentration) and neo-tissue volume on (i) image quality (i.e. image contrast), (ii) dataset quality (i.e. potential for quantitative interpretation of the obtained datasets) and (iii) tissue integrity (only for PTA). Based on the DoE outcome, optimum staining conditions for both contrast agents were selected. For these staining conditions, we were able to develop an automated, user-independent and highly quantitative image processing and analysis procedure for morphometric analysis of neo-tissue within the constructs. As a result, CE-nanoCT was optimized for routine screening of the neo-tissue formation in TE constructs transforming it to a robust quality control methodology.

## Materials and Methods

### TE constructs

#### Ti6Al4V based constructs

To evaluate the influence of the staining parameters of the contrast agents (staining time and concentration) and neo-tissue volume on the image quality, dataset quality and tissue integrity, selective laser melted porous cylindrical 3D Ti6Al4V scaffolds with a height of 6mm and a diameter of 6mm were used. The design and production of the Ti6Al4V scaffolds is described in detail in Ref. [28]. Human periosteal derived cells (hPDCs) [29] were seeded as described before using a static drop-seeding protocol [30].

In order to generate constructs with different amounts of neo-tissue they were subsequently cultured in a static setup for 3 weeks (low volumes of neo-tissue) or in an in-house developed perfusion bioreactor system for 1 to 3 weeks (respectively medium and high volumes of neo-tissue) as described earlier [21, 30]. For all conditions the constructs were cultured in growth medium (Dulbecco’s modified Eagle’s medium with high-glucose (Life Technologies) containing 10% foetal bovine serum (FBS, Gibco), 1% sodium pyruvate (Life Technologies) and 1% antibiotic—antimycotic (100 units/mL penicillin, 100 mg/mL streptomycin, and 0.25 mg/mL amphotericin B; Life Technologies)), which was refreshed every two days. For the perfusion bioreactor system a flow rate of 1 ml/min was used.

#### PCL based constructs

To assess the effect of the different contrast agents on the tissue integrity, we used porous cylindrical polycaprolactone (PCL) scaffolds with a height of 3 mm and a diameter of 6 mm. PCL scaffolds were produced using the BioscaffolderVR device (Sys-Eng, Germany). The pressure was maintained at 5 bars and the temperature was set to 120°C. The selected overlay pattern was 0–90°, the anticipated strut diameter was 100 μm and the anticipated pore size 200 μm. The scaffolds were designed in Inventor while PrimCam (Sys-Eng, Germany) was used to create the final structure [31–33]. Also the PCL scaffolds were seeded with hPDCs using a static drop-seeding protocol. The PCL based constructs were cultured using the same operating conditions in the perfusion bioreactor system as for the Ti6Al4V based constructs, but for a period of 2 weeks.

#### Contrast-enhanced nanofocus CT (CE-nanoCT)

After static or dynamic culture, the TE constructs were rinsed with 1 ml phosphate buffered saline (PBS) and transferred to a 4% paraformaldehyde solution (Sigma) for 2 hours to fixate the neo-tissue. The TE constructs were stored in PBS prior to CE-nanoCT scanning. Two contrast agents were used, namely Hexabrix 320 (Guerbet Nederland B.V) and phosphotungstic acid (PTA—VWR International). Hexabrix, containing the negatively charged ioxaglate, is an equilibrium contrast agent staining all non-negatively charged tissues. PTA on the other hand is known to specifically bind to various components of the connective tissue such as collagen and fibrin [21]. The nanoCT system used was a Phoenix NanoTom S (GE Measurement and Control Solutions).

#### Ti6Al4V constructs

For the Ti6Al4V constructs, different concentrations and staining times of both contrast agents were used according to a 3-level 3-parameter fractional factorial design as shown in Table 1. TE constructs were initially stained and scanned with Hexabrix, after which they were rinsed 3 times in PBS overnight to remove all Hexabrix traces prior to subsequent staining. The same constructs were then stained with PTA and scanned again. As after removal from the liquid prior to scanning, remnants of the staining solution could be present at locations where there is no neo-tissue, and thus could influence the visualization of the neo-tissue, samples were dried for 15 minutes on a paper tissue at room temperature for both contrast agents [21].

**Table 1:** The experimental conditions to be evaluated for the 3-level, 3-parameter fractional factorial design.

Neo-tissue volume	Staining time	Concentration
3 weeks static (Low)	120 min	60% Hex—7.5% PTA
3 weeks static (Low)	240 min	40% Hex—5% PTA
3 weeks static (Low)	30 min	20% Hex—2.5% PTA
1 week perfusion (Mid)	120 min	40% Hex—5% PTA
1 week perfusion (Mid)	240 min	20% Hex—2.5% PTA
1 week perfusion (Mid)	30 min	60% Hex—7.5% PTA
3 weeks perfusion (High)	120 min	20% Hex—2.5% PTA
3 weeks perfusion (High)	240 min	60% Hex—7.5% PTA
3 weeks perfusion (High)	30 min	40% Hex—5% PTA

For scanning the Ti6Al4V-based TE constructs, the nanoCT was equipped with a tungsten target, and was operated at a voltage of 90 kV and a current of 170 μA. A 1 mm filter of aluminum and 1 mm of copper was used to reduce beam hardening and metal artifacts as much as possible during scanning. The exposure time was 500 ms and 2400 radiographic images were acquired in fast scan mode (frame averaging of 1 and image skip of 0) resulting in a scanning time of 20 minutes per sample. The scanning time was kept low to allow routine screening and eliminate sample movement during scanning. The reconstructed images had an isotropic voxel size of 3.75 μm. A beam hardening correction of 9 and a Gaussian filter of 6 was applied during reconstruction [Datos|x, GE Measurement and Control Solutions, Germany].

#### PCL based constructs

To determine the effect of the contrast agents on the neo-tissue integrity, a comparison between the datasets of constructs with and without staining was made. Therefore, the PCL based TE constructs were consecutively scanned (1) without contrast agent, (2) with Hexabrix, (3) with PTA, (4) again with Hexabrix and finally (5) without contrast agent.

For scanning the PCL-based TE constructs, a 1 mm aluminum filter was used and the tungsten target was operated at a voltage of 60 kV and 220 μA. All other scanning as well as the reconstruction parameters were similar to what was used for the Ti6Al4V based constructs.

### DoE analysis

**Fig 1:**
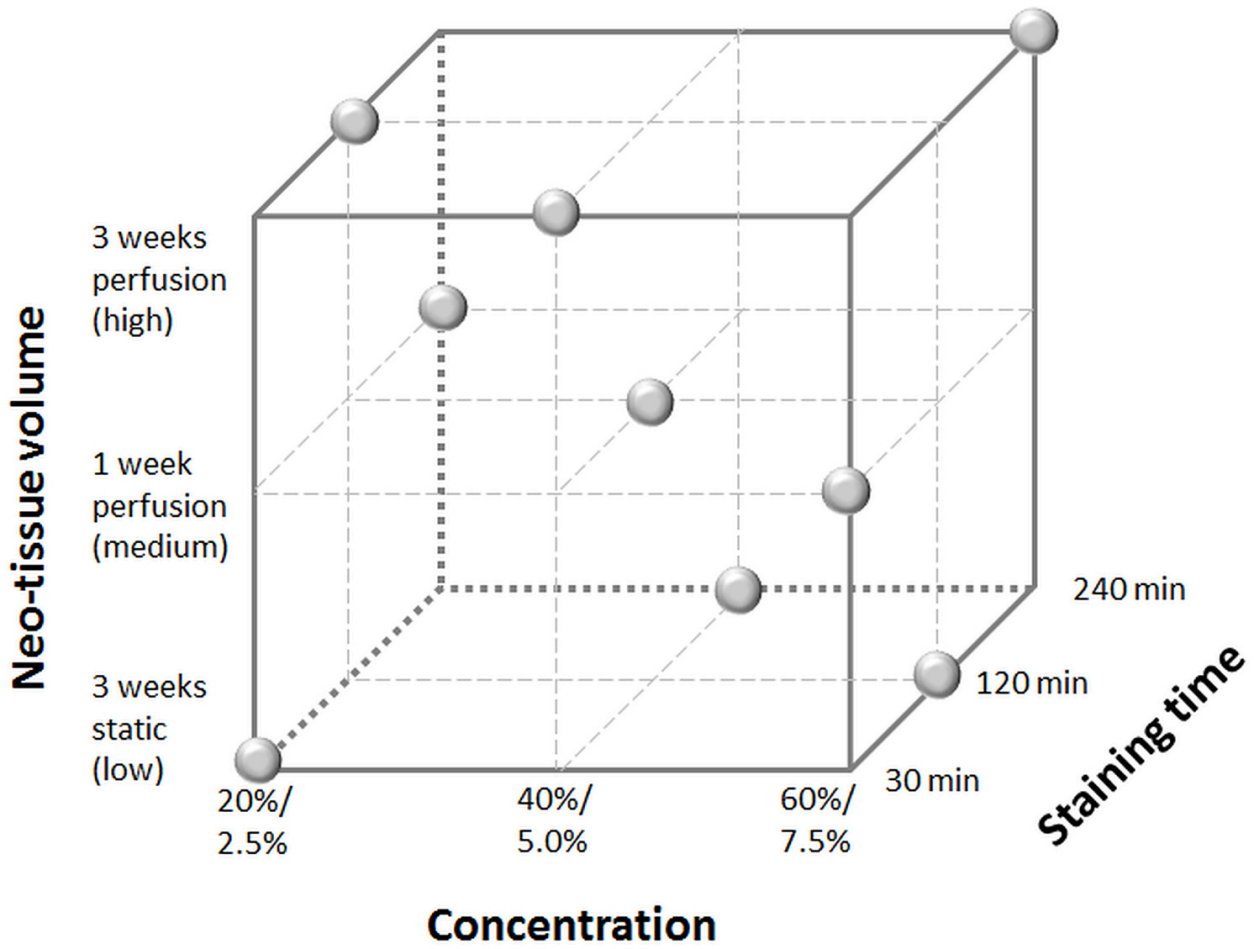
Design space for the DoE, showing the three levels of the three parameters. For the concentration, the top value is for Hexabrix staining and the bottom value for PTA staining. The grey dots indicate the experimental conditions for the fractional factorial design (n = 3).

To determine the influence of the concentration and staining time of the contrast agents as well as the neo-tissue volume (further referred to as DoE parameters) on the image quality, dataset quality and tissue integrity, a DoE approach was used. This statistical method for planning experiments enables to study the influence of various parameters with minimal required experimental input but resulting in the required objective conclusions [34]. Fig 1 and Table 1 show the different combinations of the DoE parameters, selected in a randomized manner by the statistics software JMP (SAS, Cary, USA), that were evaluated according to a 3-level, 3-factor fractional factorial design. Based on previous experiments [21] and a preliminary range screening, the three levels were selected for each parameter. Ti6Al4V based TE constructs cultured for 3 weeks in the static system represented the low neo-tissue volume level, 1 week in the perfusion bioreactor system the mid-level and 3 weeks in the perfusion bioreactor the high level [21, 30]. For each condition, 3 samples were evaluated.

DoE was applied to obtain statistically sound and objective conclusions on the magnitude and the importance of the main effects of the investigated factors (i.e. volume of neo-tissue, contrast agent concentration and staining time) on (i) the normalized contrast of the stained neo-tissue (i.e. contrast of the stained neo-tissue compared to the background and scaffold), (ii) the dataset quality (i.e. the amount of slices with a mismatch between the stained and the binarized neo-tissue fraction) and (iii) the tissue integrity (i.e. difference between the Hexabrix and PTA stained neo-tissue). The Pareto charts display the absolute values of the standardized effects and have a reference line for p = 0.05. Any effect that extends beyond this reference line is a significant effect. The more the effect extends the reference line, the more important the effect is.

#### Influence of the DoE parameters on the image contrast

**Fig 2:**
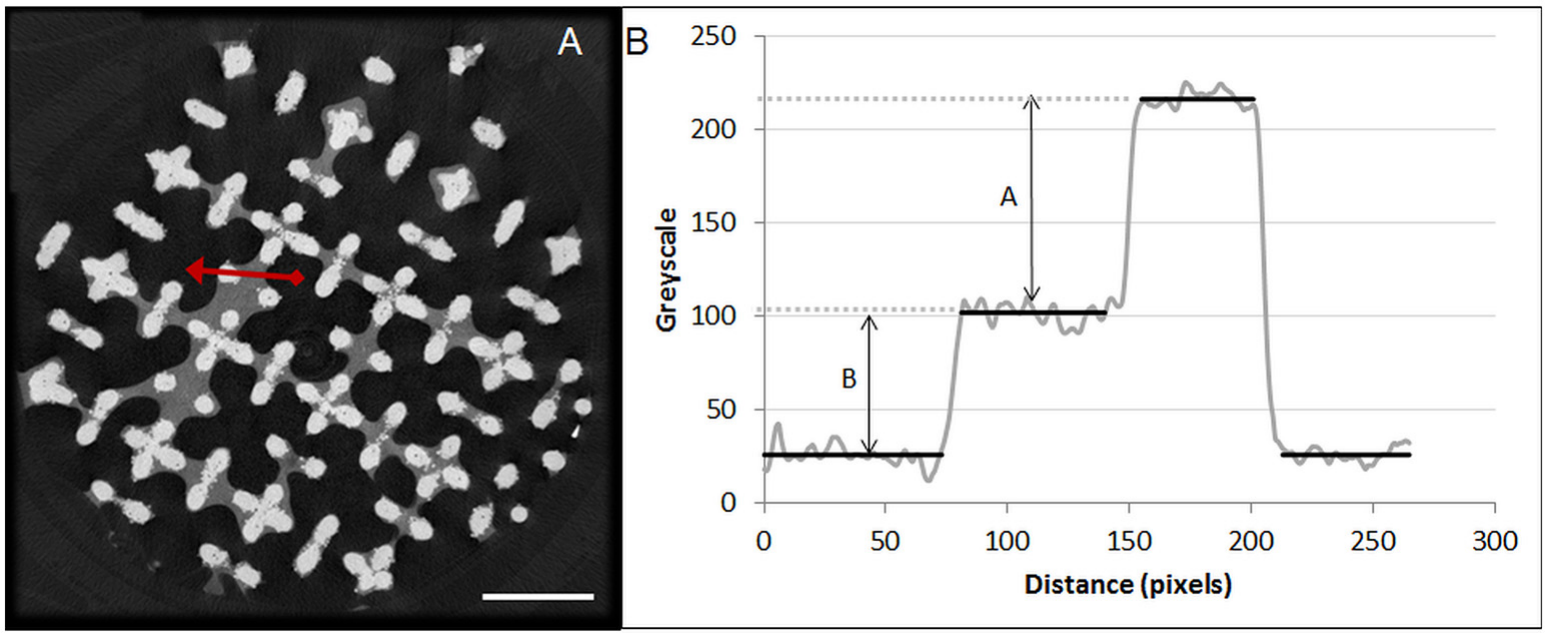
Normalized image contrast quantification of the stained neo-tissue compared to the background and scaffold. (A) A representative transaxial cross-section of a Hexabrix (60%) stained construct with a mid-level of neo-tissue volume. Scale bar is 1 mm. (B) The greyscale histogram through the red line indicated in (A), used to quantify the contrast of the stained neo-tissue compared to the background and scaffold. ‘A’ is the average greyscale difference between the stained neo-tissue and the scaffold, and ‘B’ is the average greyscale difference between the background and the stained neo-tissue.

To quantify the contrast of the stained neo-tissue compared to the background and scaffold, a greyscale histogram was plotted along an arbitrary line through the background, neo-tissue and scaffold (Fig 2A) using DataViewer (Bruker MicroCT, Belgium). The normalized contrast was then calculated according to Eq 1 (Fig 2B).

$$\frac{B}{\left(A+B\right)}=normalized\;contrast$$ (1)

where A is the average greyscale difference between the stained neo-tissue and the scaffold, and B is the average greyscale difference between the background and the stained neo-tissue. A + B is thus the average greyscale difference between the background and the scaffold. The calculated averages did not take into account the transition grey-scale values between the different phases. We only considered the grey-scale values within the range that did not deviate too much from the average value (i.e. plateau region). The thresholds to define the range for which the average was determined, were set at the grey-scale values that decreased below (or increased above) the lowest/highest value within the plateau region.

#### Influence of the DoE parameters on the dataset quality

The fraction of images containing a mismatch between the stained and binarized neo-tissue fraction, being a measure for the potential for quantitative interpretation or the dataset quality, was determined as a second input for the DoE. To obtain the binarized neo-tissue fraction multi-level Otsu segmentation was applied. This algorithm, which is a histogram-based methodology that maximizes the variance between the different classes in the greyscale image [35], was performed on each individual 2D slice using CTAn (Bruker micro-CT, Belgium). Segmentation classes corresponding to the neo-tissue were subsequently binarized. The number of levels for the Otsu segmentation had a strong influence on the mismatch, because depending on the neo-tissue volume and/or contrast, the background noise and artifacts could be assigned to different segmentation classes. Therefore, the optimal number of Otsu-levels was determined for each contrast agent based on the datasets obtained with low, mid and high levels for each DoE parameter. The optimized number of Otsu levels per contrast agent was then used on the datasets of all the DoE conditions to determine the dataset quality.

**Fig 3:**
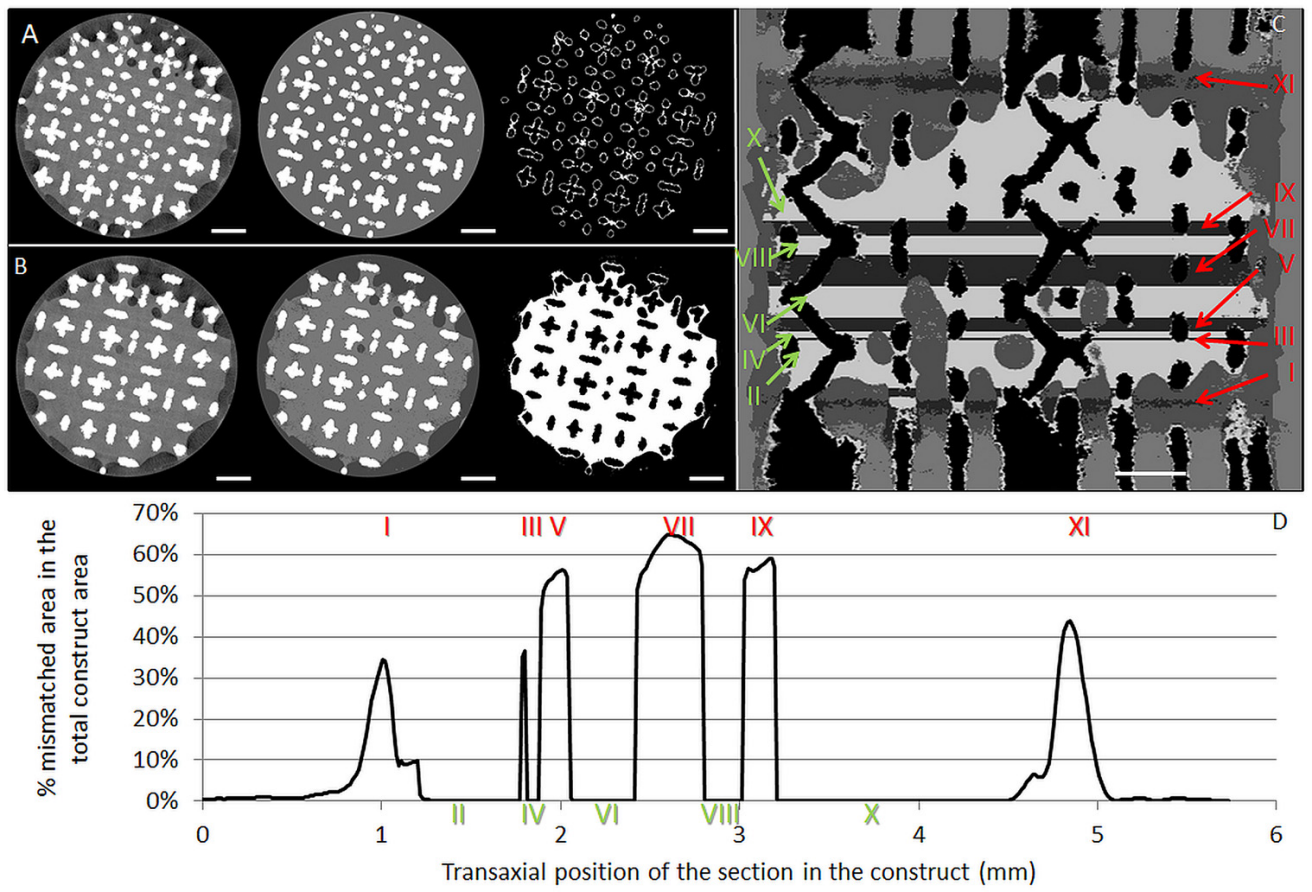
Mismatch between the stained and binarized neo-tissue fraction as a measure for the dataset quality. (A, B) A raw (left), 2-level segmented (middle) and binarized image (right) cross-section of a TE construct with a high level of neo-tissue volume after 120 min staining with 20% Hexabrix located in the middle of zone V (A) and zone VI (B). Zoning depicted in (C)—green numbers indicates zones without mismatch and red numbers indicate zones with a mismatch larger than the 1% threshold. (C) Differential overlay (coronal view) of the raw image and the binarized neo-tissue with indication of the zones (green) without mismatch between the raw and the binarized images and (red) where the 2-level segmentation resulted in a mismatch between the raw and the binarized images. (D) The histogram showing the mismatched area between the raw and the binarized images as indicated in (C). The grey striped line indicates the 1% threshold above which a mismatch was considered. Scale bars are 1 mm.

To calculate the mismatch between the stained neo-tissue and the binarized neo-tissue fraction (Fig 3A and 3B), a differential overlay of both was made (Fig 3C). Binarized differential overlays were subsequently analyzed for the ‘mismatched neo-tissue’ fraction and histograms that show the area of the mismatched fraction were generated (Fig 3D). Based on these histograms, the fraction of mismatched images was determined. In order to omit the influence of background noise on the analysis, slides with a mismatch lower than 1% of the total slide surface were excluded from the analysis.

#### Influence of the DoE parameters for PTA staining on the neo-tissue integrity

To first evaluate the influence of both contrast agents on the neo-tissue integrity, a comparison between the datasets of constructs with and without staining was made. For the Ti6Al4V-based TE constructs, visualization of the neo-tissue without contrast agent was not possible as the scanning energies required to obtain sufficient transmission through the metal scaffold resulted in greyscale values for the neo-tissue that could not be segmented from the background noise and streaks. Therefore, PCL based TE constructs were used, as the lower X-ray opacity of PCL enabled the visualization of the neo-tissue without the presence of a contrast agent. Differential overlays of the datasets of TE constructs consecutively scanned (1) without contrast agent, (2) with Hexabrix, (3) with PTA, (4) again with Hexabrix and finally (5) without contrast agent were generated. The mismatch between the binarized neo-tissue for the different scans was determined to evaluate the influence of the contrast agent on the tissue integrity, and the potential staining-induced tissue shrinkage.

**Fig 4:**
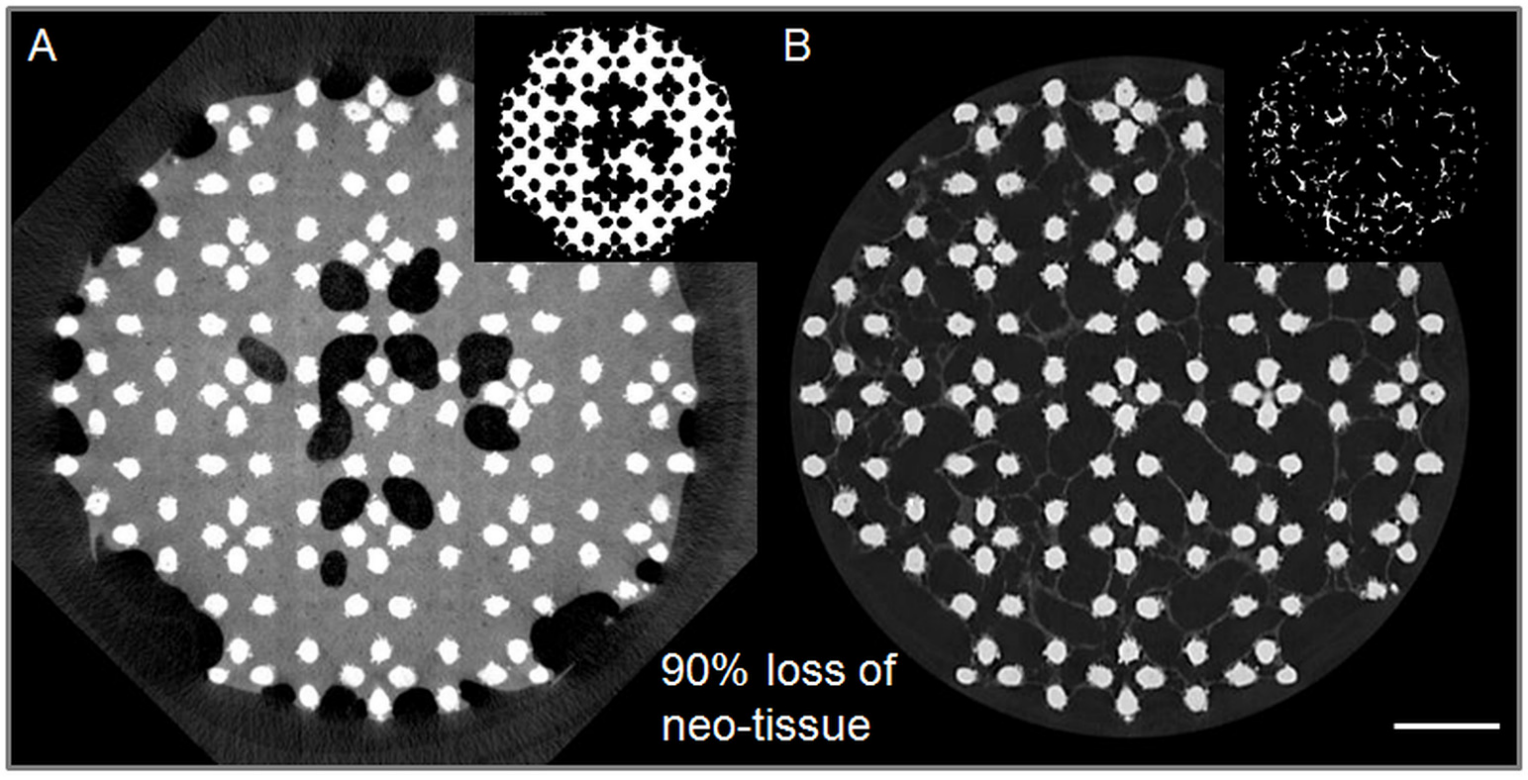
Quantification of tissue shrinkage. Representative transaxial cross-sections of (A) a Hexabrix (60%, 240 min) stained TE construct and (B) the same TE construct stained with PTA (7.5%, 240 min). The insets show the binarized images of the neo-tissue in the cross-section, based on which the surface area of neo-tissue was calculated. Scale bar is 1 mm.

Then, as final DoE read-out, the influence of the PTA staining on the tissue integrity was determined. This was quantified as the difference between the Hexabrix (Fig 4A) and PTA (Fig 4B) stained neo-tissue surface area relative to the Hexabrix stained neo-tissue surface area in the corresponding CE-nanoCT slices. Hereto, the Hexabrix and PTA stained datasets of the same sample were registered in 3D using DataViewer (Bruker MicroCT, Belgium). To avoid the evaluation of mismatched slices, three transaxial slices per dataset, distributed over the height of the TE construct, were selected. Using the optimal segmentation procedure for the different contrast agents as described earlier, the neo-tissue was binarized applying CTAn (Bruker MicroCT, Belgium). To reduce the errors introduced by the partial volume effect (PVE) and metallic artifacts for analyzing the neo-tissue surface area, the binarized images for the scaffold structure were dilated by 2 voxels and subtracted from the dataset of the binarized neo-tissue. The noise in the binarized neo-tissue images was minimized by removing black and white speckle noise smaller than 200 voxels. In order to solidify the resulting structure, a ‘closing’ operation of 2 voxels was performed. Finally, the surface area of the binarized neo-tissue was quantified and the degree of tissue shrinkage (i.e. inverse of the tissue integrity) was determined relative to the Hexabrix stained neo-tissue using Eq 2. The average neo-tissue thickness was calculated on every CT slice using the 2D thickness analysis algorithm from CTAn (Bruker MicroCT). Based on the 3D thickness analysis ona subsection without mismatch of the datasets, 3D color-coded rendering showing the neo-tissue thickness distribution were generated using CTVox (Bruker MicroCT).

$$\frac{SA_{Hex}-SA_{PTA}}{SA_{Hex}}=de\;gree\;of\;tissue\;shrinkage$$ (2)

where SA_Hex_ is the Hexabrix stained neo-tissue surface area and SA_PTA_ is the PTA stained neo-tissue surface area in the corresponding CE-nanoCT slice.

## Results and Discussion

### Image quality

**Fig 5:**
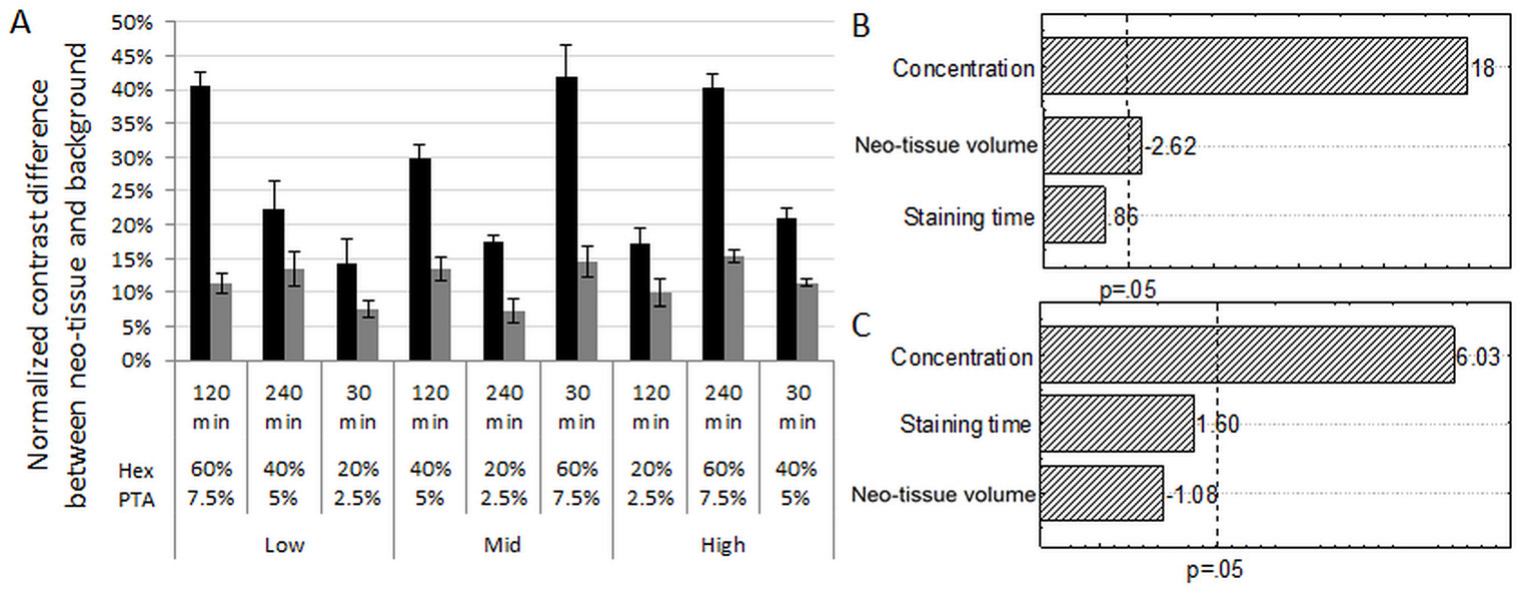
Influence of all DoE parameters on normalized contrast. (A) Normalized contrast difference between neo-tissue and background as shown in Eq 1 for Hexabrix (black) and PTA (grey) for all conditions of the DoE. Pareto charts for (B) Hexabrix and (C) PTA stained TE constructs, showing the ranking of the influence of all staining parameters on normalized contrast. The dotted line indicates a significance level of p = 0.05.

As a DoE analysis read-out, the normalized contrast of the stained neo-tissue compared to the background and scaffold was determined for each experimental condition. For both contrast agents, the concentration was the most influencing parameter (Fig 5B and 5C). This could also be observed from the raw data (Fig 5A), which showed a concentration-dependent increase in relative contrast, independent of the staining time or neo-tissue volume.

Staining time did not significantly influence the normalized contrast, indicating that both contrast agents had infiltrated the neo-tissue within 30 minutes, even for the largest neo-tissue volume (Fig 5B and 5C). Although limitations in staining homogeneity and intensity due to diffusion limitations of the contrast agent have been reported [22], these were not observed in our experiments. Non-mineralized neo-tissue developed in a perfusion bioreactor system, such as the neo-tissue in this study, was reported to have an approximate cell density of 6 * 10^6^ cells/cm^3^ [30, 36], while the density of native tissue, in which diffusion limitations were previously reported, can on average be between 10^8^ and 10^9^ cells/cm^3^. Since no influence of the staining time was observed within the operating window of our experiments, the lower cell density of the engineered neo-tissues will enable both contrast agents to fully infiltrate within the minimal staining time of 30 min.

For Hexabrix (Fig 5B), the normalized neo-tissue contrast was negatively influenced by the neo-tissue volume. As shown in earlier work, the neo-tissue has a fibrous structure [37]. Due to the limited spatial image resolution and the dense packing of the individual fibers (i.e. inter-fiber distances below the spatial image resolution), they cannot be discriminated in the CE-nanoCT images. Moreover, since Hexabrix is an equilibrium contrast agent, the staining solution might be entrapped between neo-tissue fibers during infiltration. For the high level of neo-tissue volume, the neo-tissue fibers might be more densely packed and we could hypothesize that less volume is available within the porous neo-tissue for the Hexabrix solution to be entrapped. As a result, the greyscale of the bulk neo-tissue will be lower compared to the less packed neo-tissue for the low- and mid-level of neo-tissue volume. Consequently, the normalized contrast of the neo-tissue is lower, as depicted in the pareto chart.

### Dataset quality

#### Optimization of the image segmentation procedure

**Fig 6:**
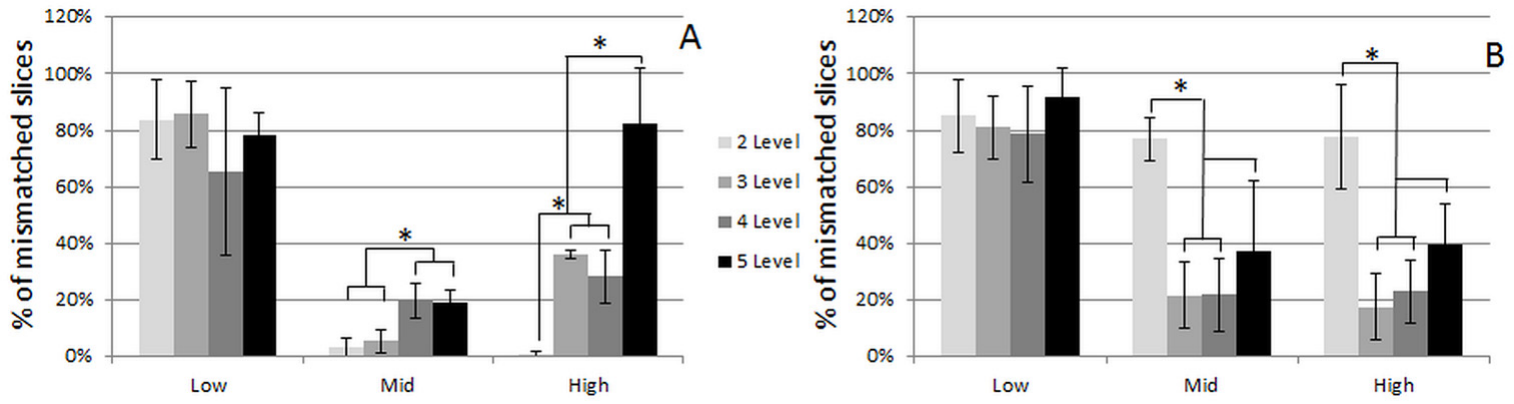
Percentage of mismatched slices for different Otsu segmentation levels and for all DoE parameters. Percentage of slices of the full dataset showing a mismatch between the stained and binarized neo-tissue for different Otsu segmentation levels and different value sets for all three DoE parameters (neo-tissue volume, staining time and concentration) for (A) Hexabrix and (B) PTA stained TE constructs. *p < 0.05.

To quantitatively and objectively evaluate which segmentation procedure was optimal for each contrast agent, the fraction of images containing a mismatch between the stained and the binarized neo-tissue fraction was determined for datasets with low, mid and high levels of each DoE factor. Fig 6 indicates that both for the Hexabrix and PTA stained ‘low-level’ constructs (low values of all 3 parameters), binarization of the neo-tissue could not be done correctly, hence excluding automated analysis of the neo-tissue volume for these conditions. Both for the Hexabrix-stained TE constructs with a mid- and high-level parameter set, the 2-level Otsu segmentation enabled binarization of the neo-tissue with a minor mismatch (Fig 6A), and was therefore further used for the evaluation of the Hexabrix stained TE constructs. For the PTA stained TE constructs with a mid- and high-level neo-tissue volume, all segmentation levels resulted in a high mismatch between the stained and binarized neo-tissue fraction. No significant differences were observed between the 3, 4 and 5-level segmented images (Fig 6B). However, the more levels that needed to be segmented, the larger the processing time that was required to generate the segmented images. Therefore a 3-level Otsu segmentation was used for further analysis of the PTA-stained TE constructs.

#### Influence of DoE parameters on the dataset quality

**Fig 7:**
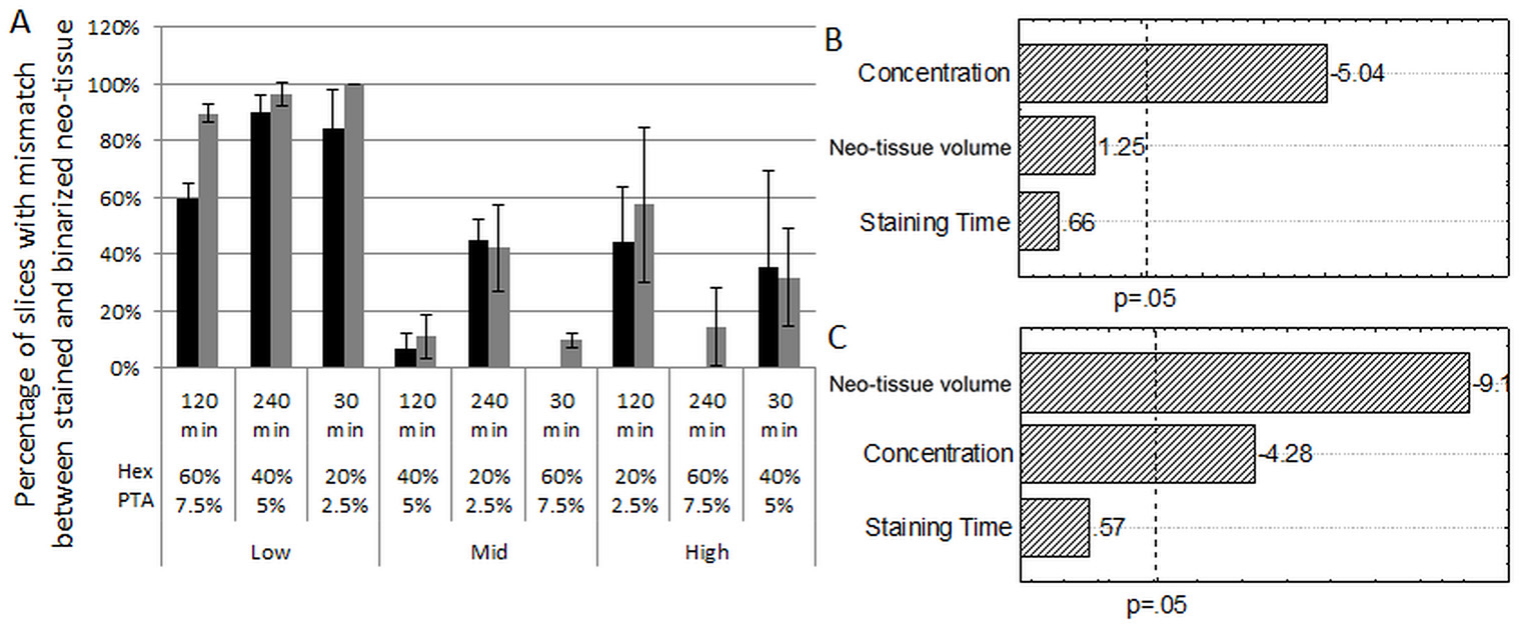
Influence of all DoE parameters on the mismatch between the stained and binarized neo-tissue. (A) The percentage of slices with a mismatch between the stained and binarized neo-tissue for Hexabrix (black) and PTA (grey) for all conditions of the DoE. (B, C) Pareto charts for Hexabrix (B) and PTA (C) stained TE constructs respectively, showing the influence of the different staining parameters on the mismatch. The dotted line indicates a significance level of p = 0.05.

To determine the influence of the DoE parameters on the dataset quality, the fraction of slices with a mismatch between the stained and binarized neo-tissue was evaluated using the optimized segmentation settings as described above. For both contrast agents, their concentration significantly influenced the fraction of mismatched slices (Fig 7B and 7C). As the normalized contrast of neo-tissue increased with increasing concentration, it could be more easily separated from the scaffold-dependent image artifacts, and thus the amount of mismatched slices decreased significantly (Fig 7A).

**Fig 8:**
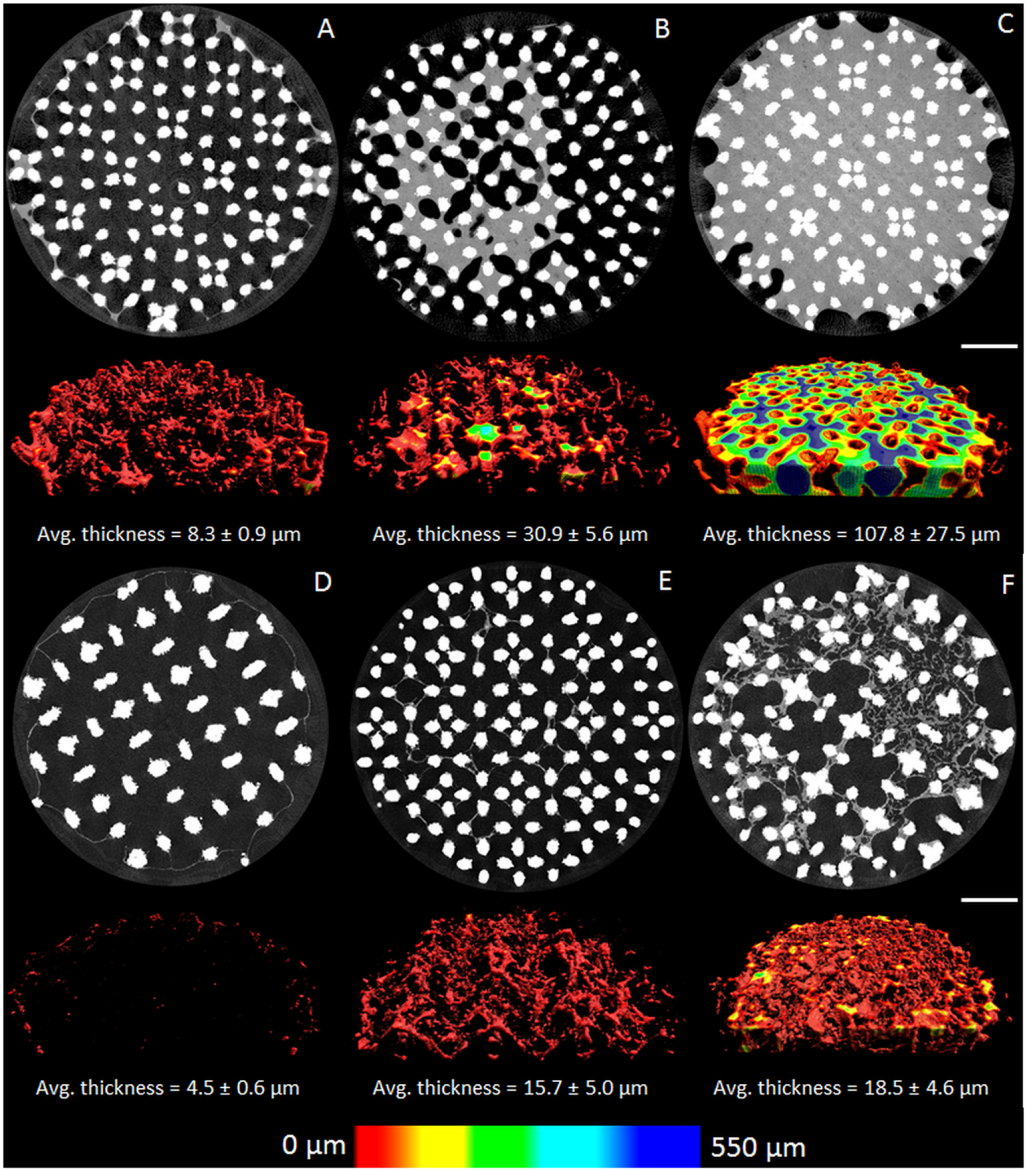
Morphology and thickness of the neo-tissue for different DoE conditions. (A-C) Representative cross-sectional images and 3D color-coded renderings for TE constructs with (from left to right) low, medium and high levels of neo-tissue volume, stained with 60% Hexabrix. (D-F) Representative cross-sectional images and 3D color-coded renderings for TE constructs with (from left to right) low, medium and high levels of neo-tissue volume, stained with 7.5% PTA. Scale bar is 1 mm. For each condition, the average neo-tissue thickness is included (n = 3).

Both contrast agents showed a high mismatch for low volumes of neo-tissue (Fig 7A). This confirmed the findings from our previous study, where we determined the CE-nanoCT threshold for neo-tissue quantification to be a neo-tissue volume lower than 4% of the total TE construct void volume [21]. This lower quantification limit was directly related to the artifacts introduced by the metallic scaffolds. The DoE analysis showed however that only in the case of PTA the neo-tissue volume had a significant effect on the dataset quality, as an increase in neo-tissue volume resulted in a significant decrease in mismatched slices (Fig 7C). This is mainly caused by the neo-tissue structure, which for the low neo-tissue volume is on average much thinner compared to the other levels of neo-tissue volume (Fig 8). When compared to the Hexabrix stained neo-tissue (Fig 8), the PTA stained neo-tissue showed for all experimental conditions a more fibrous structure. Moreover its thickness could approach the spatial resolution of the images (i.e. 7.5 μm–11.5 μm). Consequently, the PVE reduces the contrast of the neo-tissue, making it no longer separable from the scaffold-dependent image artifacts. Additionally, when the volume fraction of the bulk neo-tissue became too small, as was the case for the low neo-tissue volume, its peak in the cross-section greyscale histogram could no longer be segmented from the other phases in the images. The combination of the above mentioned factors all contributed to the mismatch quantified in Fig 7.

### Tissue integrity

#### Influence of the different contrast agents on the neo-tissue integrity

**Fig 9:**
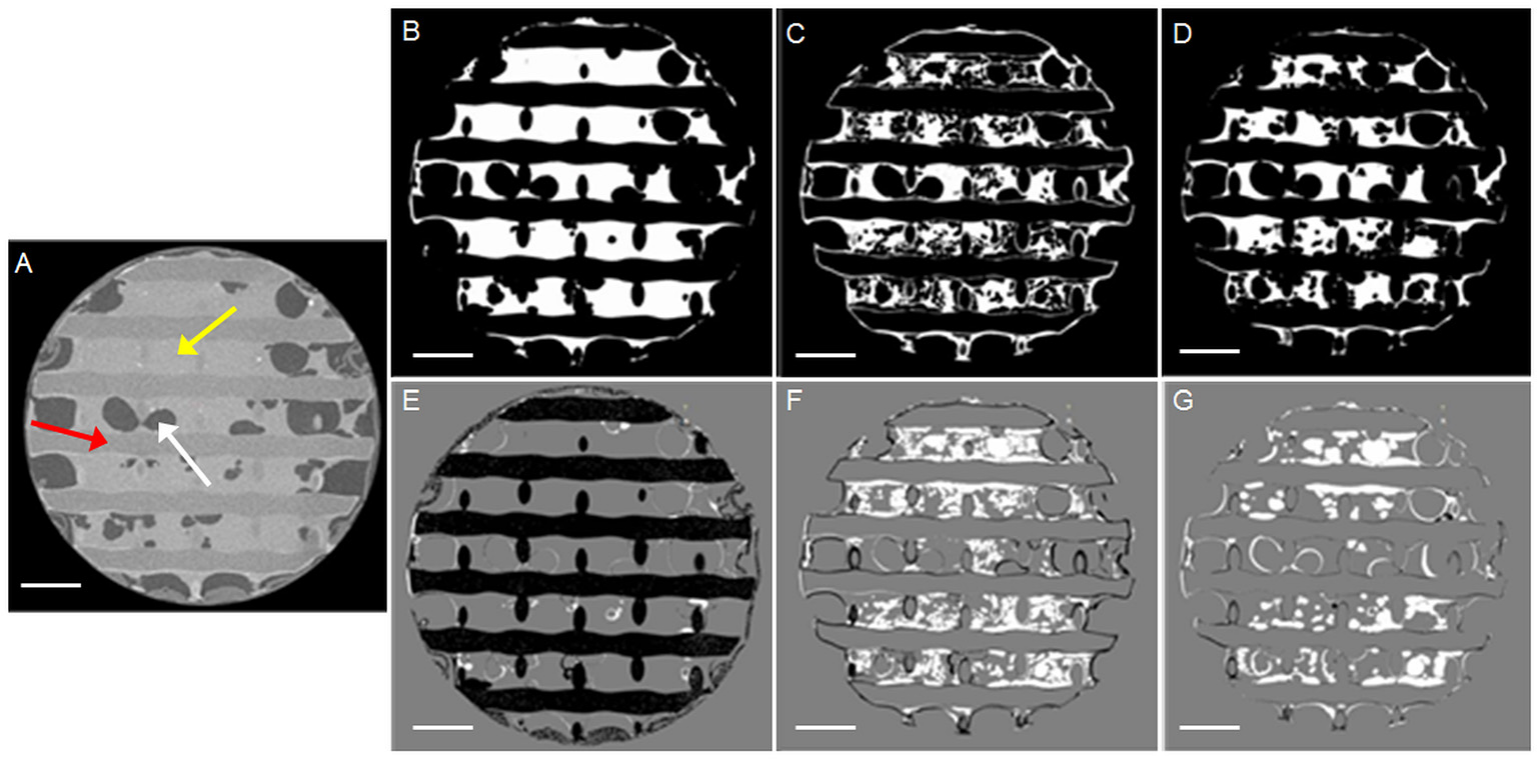
Influence of the different contrast agents on the neo-tissue integrity. (A) A typical transaxial cross-section through a PCL-based TE construct without a contrast agent. The red arrow indicates the PCL scaffold; the yellow arrow indicates the neo-tissue; the white arrow indicates the air space. The corresponding binarized images for neo-tissue of the (B) Hexabrix (60%) stained dataset, (C) PTA (7.5%) stained dataset after Hexabrix staining and (D) Hexabrix (60%) stained dataset after the consecutive Hexabrix and PTA staining. (E) The differential overlay image of (A) and (B); (F) the differential overlay image of (B) and (C); and (G) differential overlay image of (B) and (D). The black voxels in the overlay images represent the voxels present in the first dataset, but not in the second; the white voxels represent the voxels present in the second dataset, but not in the first. Scale bars are1 mm.

As mentioned earlier, the use of PTA as a contrast agent can cause tissue shrinkage [24]. For Hexabrix, as this is a clinically approved contrast agent that does not chemically bind to the tissue, no effect on the tissue integrity was expected. To the best of our knowledge, there are no studies in the literature that have reported an effect of Hexabrix staining on the tissue integrity. Using PCL-based TE constructs without and with Hexabrix staining (Fig 9A and 9B respectively), we could confirm this, as the PCL scaffolds allow us to visualize the neo-tissue without the use of a contrast agent. When overlaying both datasets (Fig 9E), the largest difference (indicated in black) was the scaffold itself, since this could not be segmented from the neo-tissue in the images without contrast agent. Some small differences (indicated in white) were found in the small pores, where the Hexabrix solution might have been not removed during the short drying step prior to scanning (on average 4.81 ± 3.95% of the total neo-tissue volume, n = 3). The viscosity of the Hexabrix solution is higher than that of the PBS itself, causing a more difficult removal of the liquid from the pores during the drying step. We could therefore conclude that the Hexabrix staining does not influence the neo-tissue structure.

Subsequent to the Hexabrix staining and an overnight rinsing step in PBS solution to remove the Hexabrix, TE constructs were stained with PTA and imaged again. A strong influence on the neo-tissue integrity was appreciated (Fig 9C), and a clear difference with the Hexabrix stained dataset was seen (Fig 9F). To confirm that this difference was due to the PTA staining, the TE construct was once again stained with Hexabrix after overnight rinsing in PBS and scanned (Fig 9D). Although the small pores were again filled with the Hexabrix solution as mentioned earlier, a significant difference was seen compared to the first Hexabrix stained dataset (up to 29 ± 17.5% of the total neo-tissue volume), indicating that the PTA staining indeed caused tissue shrinkage.

#### Influence of DoE parameters for PTA staining on the neo-tissue integrity

**Fig 10:**
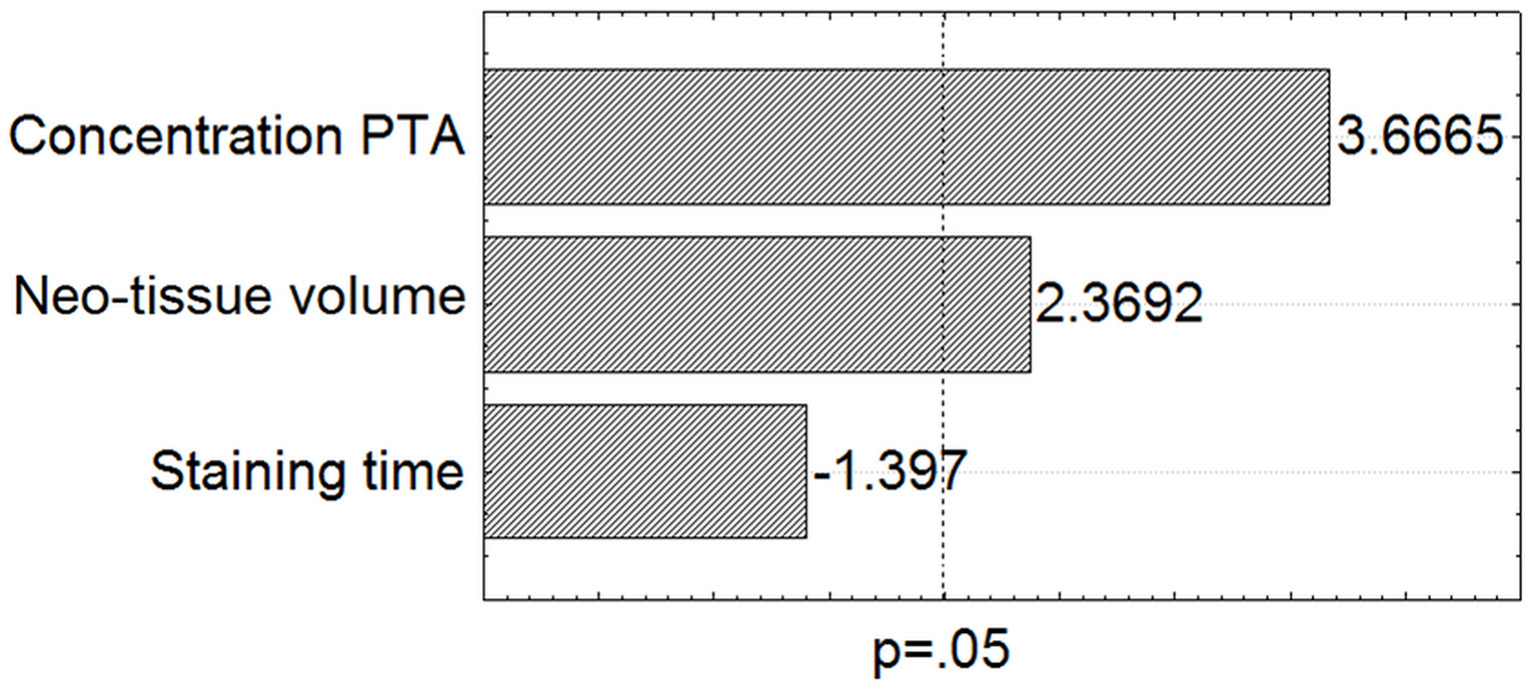
Influence of the DoE parameters for PTA staining on the neo-tissue integrity. Pareto chart for the PTA-stained TE constructs when evaluating the degree of tissue shrinkage. The dotted line indicates a significance level of p = 0.05.

The degree of tissue shrinkage due to PTA staining was quantified as the difference between the Hexabrix and PTA stained neo-tissue surface area in the corresponding CE-nanoCT slices relative to the Hexabrix stained neo-tissue surface area. In agreement with other studies [23, 24], the DoE showed that the concentration of the contrast agents has the strongest effect on the tissue shrinkage (Fig 10). Although an increase in the concentration caused a significant increase in the normalized neo-tissue contrast (Fig 5C), as well as a significant decrease in the amount of mismatched images (Fig 7C), it did introduce a significant tissue shrinkage. Hence, when accurate neo-tissue morphometric analyses should be performed, the concentration should be kept as low as possible. Additionally, also an increase in neo-tissue volume was shown to result in a significant increase in tissue shrinkage, which could be expected as more neo-tissue is available for the PTA to affect.

As the chemistry of the staining is not fully defined yet [23], we cannot give a clear explanation for the tissue shrinkage. It has however been shown that tissue shrinkage because of the contrast agents is strongly dependent on the tissue composition and structure, since a different degree of shrinkage was found for different soft and mineralized tissue types [21, 23, 24]. As we currently have no quantitative data on the composition or fibrous structure of the neo-tissue for the different levels of neo-tissue volume, we cannot claim that this could influence the tissue shrinkage. Histological sectioning would have enabled us to confirm the fibrous structure of the neo-tissue. Although we did attempt to make histological sections of the PCL scaffolds post scanning using various embedding techniques such as paraffin, OCT (optimal cutting temperature compound) and PMMA (polymethylmethacrylate) embedding, this was without success. Sectioning of these constructs resulted in severe cutting artifacts, which made histological analysis of neo-tissue structure impossible. We did however show using CE-nanoCT that the bulk neo-tissue structure for the low-level neo-tissue volume was much thinner than for the mid- and high-level neo-tissue volume. As a result, the segmentation and binarization of the neo-tissue was less accurate (Fig 7). Because of this, and due to the influence of the PVE, the calculation of the degree of tissue shrinkage for the low-level of the neo-tissue volume might be inaccurate and should be evaluated with care.

## Conclusions

Using a DoE approach, we were able to provide the most optimal staining conditions for CE-nanoCT of neo-tissue in Ti6Al4V scaffolds by quantifying and ranking in statistical importance the factors that influence the CE-nanoCT staining procedure using Hexabrix and PTA. These factors were contrast agent concentration, staining time and the presence of neo-tissue (volume). Their effect on image quality, dataset quality and tissue integrity was investigated.

For Hexabrix staining of the neo-tissue in Ti alloy scaffolds, in this study the most optimal staining conditions were (i) a concentration of 60% (vol% in PBS) and (ii) a staining time of 30 minutes. These were sufficient to allow accurate and fully automated quantification of the neo-tissue formed. When the neo-tissue volume fraction was however less than 4% of the open space, in this study represented by the low-level neo-tissue volume, quantitative analysis of the datasets was not possible.

For PTA staining, the DoE showed that in this study a high concentration was necessary for sufficient image contrast and accurate, automated 2D/3D image analysis, although significant tissue shrinkage was introduced. Hence, based on the results from the DoE, for this study we concluded using only 2.5% (weight% in PBS) of PTA. As the staining time did not have any significant influence, this was kept as low as possible (in this case 30 minutes).

As a result of this study, and by using a DoE approach to optimize the staining conditions, we have converted CE-nanoCT into a quantitative and 3D measuring tool to evaluate neo-tissue formation in TE constructs and its spatial distribution.

## References

[1] Eniwumide JO, Yuan H, Cartmell SH, Meijer GJ, de Bruijn JD. Ectopic bone formation in bone marrow stem cell seeded calcium phosphate scaffolds as compared to autograft and (cell seeded) allograft. Eur Cells Mater. 2007;14:30–8. . 10.22203/ecm.v014a03 17674330

[2] Hedberg EL, Kroese-Deutman HC, Shih CK, Lemoine JJ, Liebschner MAK, Miller MJ, et al Methods: A comparative analysis of radiography, microcomputed tomography, and histology for bone tissue engineering. Tissue Engineering. 2005;11(9–10):1356–67. . 16259591 10.1089/ten.2005.11.1356

[3] Smith LE, Smallwood R, Macneil S. A comparison of imaging methodologies for 3D tissue engineering. Microsc Res Tech. 2010;73(12):1123–33. Epub 2010/10/29. 10.1002/jemt.20859 . 20981758

[4] Nam SY, Ricles LM, Suggs LJ, Emelianov SY. Imaging Strategies for Tissue Engineering Applications. Tissue Eng Part B Rev. 2014 Epub 2014/07/12. 10.1089/ten.TEB.2014.0180 . 25012069 PMC4322020

[5] Jones AC, Arns CH, Sheppard AP, Hutmacher DW, Milthorpe BK, Knackstedt MA. Assessment of bone ingrowth into porous biomaterials using MICRO-CT. Biomaterials. 2007;28(15):2491–504. . 17335896 10.1016/j.biomaterials.2007.01.046

[6] van Lenthe GH, Hagenmuller H, Bohner M, Hollister SJ, Meinel L, Muller R. Nondestructive micro-computed tomography for biological imaging and quantification of scaffold-bone interaction in vivo. Biomaterials. 2007;28(15):2479–90. . 17258316 10.1016/j.biomaterials.2007.01.017

[7] Davis TJ, Gao D, Gureyev TE, Stevenson AW, Wilkins SW. Phase-Contrast Imaging of Weakly Absorbing Materials Using Hard X-Rays. Nature. 1995;373(6515):595–8. 10.1038/373595a0 .

[8] Wilkins SW, Nesterets YI, Gureyev TE, Mayo SC, Pogany A, Stevenson AW. On the evolution and relative merits of hard X-ray phase-contrast imaging methods. Philosophical transactions Series A, Mathematical, physical, and engineering sciences. 2014;372(2010):20130021 Epub 2014/01/29. 10.1098/rsta.2013.0021 . 24470408

[9] Langer M, Liu Y, Tortelli F, Cloetens P, Cancedda R, Peyrin F. Regularized phase tomography enables study of mineralized and unmineralized tissue in porous bone scaffold. Journal of microscopy. 2010;238(3):230–9. Epub 2010/06/29. 10.1111/j.1365-2818.2009.03345.x . 20579261

[10] Appel A, Anastasio MA, Brey EM. Potential for imaging engineered tissues with X-ray phase contrast. Tissue Eng Part B Rev. 2011;17(5):321–30. Epub 2011/06/21. 10.1089/ten.TEB.2011.0230 21682604 PMC3179620

[11] Voronov RS, Vangordon SB, Shambaugh RL, Papavassiliou DV, Sikavitsas VI. 3D Tissue Engineered Construct Analysis via Conventional High Resolution MicroCT without X-Ray Contrast. Tissue Eng Part C Methods. 2012 Epub 2012/10/02. 10.1089/ten.TEC.2011.0612 . 23020551

[12] Xie L, Lin ASP, Guldberg RE, Levenston ME. Nondestructive assessment of sGAG content and distribution in normal and degraded rat articular cartilage via EPIC-mu CT. Osteoarthritis and Cartilage. 2010;18(1):65–72. 10.1016/j.joca.2009.07.014 . 19744590 PMC3268049

[13] Lakin BA, Grasso DJ, Shah SS, Stewart RC, Bansal PN, Freedman JD, et al Cationic agent contrast-enhanced computed tomography imaging of cartilage correlates with the compressive modulus and coefficient of friction. Osteoarthritis Cartilage. 2013;21(1):60–8. Epub 2012/10/09. 10.1016/j.joca.2012.09.007 23041438 PMC3878721

[14] Kerckhofs G, Sainz J, Maréchal M, Wevers M, Van de Putte T, Geris L, et al Contrast-Enhanced Nanofocus X-Ray Computed Tomography Allows Virtual Three-Dimensional Histopathology and Morphometric Analysis of Osteoarthritis in Small Animal Models. Cartilage. 2014;5(1):55–65. 26069685 10.1177/1947603513501175 PMC4297096

[15] Kerckhofs G, Sainz J, Wevers M, Van de Putte T, Schrooten J, TiGenix N. Contrast-enhanced nanofocus computed tomography images the cartilage subtissue architecture in three dimensions. European Cells and Materials. 2013;25:179–89. 23389752 10.22203/ecm.v025a13

[16] Fei J, Peyrin F, Malaval L, Vico L, Lafage-Proust MH. Imaging and quantitative assessment of long bone vascularization in the adult rat using microcomputed tomography. Anat Rec (Hoboken). 2010;293(2):215–24. Epub 2009/12/04. 10.1002/ar.21054 . 19957340

[17] Granton PV, Pollmann SI, Ford NL, Drangova M, Holdsworth DW. Implementation of dual- and triple-energy cone-beam micro-CT for postreconstruction material decomposition. Med Phys. 2008;35(11):5030–42. Epub 2008/12/17. . 19070237 10.1118/1.2987668

[18] Lusic H, Grinstaff MW. X-ray-computed tomography contrast agents. Chemical reviews. 2013;113(3):1641–66. Epub 2012/12/06. 10.1021/cr200358s 23210836 PMC3878741

[19] Metscher BD. MicroCT for developmental biology: a versatile tool for high-contrast 3D imaging at histological resolutions. Dev Dyn. 2009;238(3):632–40. Epub 2009/02/25. 10.1002/dvdy.21857 . 19235724

[20] Wong MD, Dorr AE, Walls JR, Lerch JP, Henkelman RM. A novel 3D mouse embryo atlas based on micro-CT. Development. 2012;139(17):3248–56. Epub 2012/08/09. 10.1242/dev.082016 . 22872090 PMC6514304

[21] Papantoniou I, Sonnaert M, Geris L, Luyten FP, Schrooten J, Kerckhofs G. Three-dimensional characterization of tissue-engineered constructs by contrast-enhanced nanofocus computed tomography. Tissue Eng Part C Methods. 2014;20(3):177–87. Epub 2013/06/27. 10.1089/ten.TEC.2013.0041 23800097 PMC3936499

[22] Pauwels E, Van Loo D, Cornillie P, Brabant L, Van Hoorebeke L. An exploratory study of contrast agents for soft tissue visualization by means of high resolution X-ray computed tomography imaging. Journal of microscopy. 2013;250(1):21–31. Epub 2013/02/26. 10.1111/jmi.12013 . 23432572

[23] Vickerton P, Jarvis J, Jeffery N. Concentration-dependent specimen shrinkage in iodine-enhanced microCT. Journal of Anatomy. 2013;223(2):185–93. 10.1111/Joa.12068 . 23721431 PMC3724211

[24] Buytaert JAN, Johnson SB, Dierick M, Salih WHM, Santi PA. MicroCT versus sTSLIM 3D Imaging of the Mouse Cochlea. Journal of Histochemistry & Cytochemistry. 2013;61(5):382–95. 10.1369/0022155413478613 . 23360693 PMC3636707

[25] Schulz-Mirbach T, Hess M, Metscher BD. Sensory epithelia of the fish inner ear in 3D: studied with high-resolution contrast enhanced microCT. Frontiers in zoology. 2013;10(1):63 Epub 2013/10/29. 10.1186/1742-9994-10-63 24160754 PMC4177137

[26] Jeffery NS, Stephenson RS, Gallagher JA, Jarvis JC, Cox PG. Micro-computed tomography with iodine staining resolves the arrangement of muscle fibres. J Biomech. 2011;44(1):189–92. Epub 2010/09/18. 10.1016/j.jbiomech.2010.08.027 . 20846653

[27] Metscher BD. MicroCT for comparative morphology: simple staining methods allow high-contrast 3D imaging of diverse non-mineralized animal tissues. BMC Physiol. 2009;9:11 Epub 2009/06/24. 1472-6793-9-11 [pii] 10.1186/1472-6793-9-11 19545439 PMC2717911

[28] Van Bael S, Kerckhofs G, Moesen M, Pyka G, Schrooten J, Kruth JP. Micro-CT-based improvement of geometrical and mechanical controllability of selective laser melted Ti6Al4V porous structures. Mat Sci Eng a-Struct. 2011;528(24):7423–31. 10.1016/j.msea.2011.06.045 .

[29] De Bari C, Dell'Accio F, Vanlauwe J, Eyckmans J, Khan IM, Archer CW, et al Mesenchymal multipotency of adult human periosteal cells demonstrated by single-cell lineage analysis. Arthritis Rheum. 2006;54(4):1209–21. Epub 2006/04/01. 10.1002/art.21753 . 16575900

[30] Sonnaert M, Papantoniou I, Bloemen V, Kerckhofs G, Luyten FP, Schrooten J. Human periosteal-derived cell expansion in a perfusion bioreactor system: proliferation, differentiation and extracellular matrix formation. Journal of tissue engineering and regenerative medicine. 2014 Epub 2014/09/05. 10.1002/term.1951 . 25186024

[31] Berneel E, Desmet T, Declercq H, Dubruel P, Cornelissen M. Double protein-coated poly-epsilon-caprolactone scaffolds: Successful 2D to 3D transfer. J Biomed Mater Res Part A. 2012;100A(7):1783–91. 10.1002/jbm.a.34125 . 22488989

[32] Berneel EM, Desmet T, Declercq H, Dubruel P, Cornelissen R. Biological evaluation of a successful transferred double protein coating from 2D PCL coatings to 3D scaffolds. Journal of Tissue Engineering and Regenerative Medicine. 2012;6:372-. .

[33] Desmet T, Billiet T, Berneel E, Cornelissen R, Schaubroeck D, Schacht E, et al Post-Plasma Grafting of AEMA as a Versatile Tool to Biofunctionalise Polyesters for Tissue Engineering. Macromol Biosci. 2010;10(12):1484–94. 10.1002/mabi.201000147 . 20857390

[34] Kreutz C, Timmer J. Systems biology: experimental design. Febs Journal. 2009;276(4):923–42. 10.1111/j.1742-4658.2008.06843.x . 19215298

[35] Otsu N. Threshold Selection Method from Gray-Level Histograms. Ieee T Syst Man Cyb. 1979;9(1):62–6. .

[36] Sonnaert M, Papantoniou I, Luyten FP, Schrooten J. Quantitative validation of the Presto Blue metabolic assay for on-line monitoring of cell proliferation in a 3D perfusion bioreactor system. Tissue Eng Part C Methods. 2014 Epub 2014/10/23. 10.1089/ten.TEC.2014.0255 . 25336207 PMC4442584

[37] Papantoniou I, Chai YC, Luyten FP, Schrooten J. Process quality engineering for bioreactor-driven manufacturing of tissue engineered constructs for bone regeneration. Tissue Eng Part C Methods. 2013;19(8):596–609. Epub 2012/12/04. 10.1089/ten.TEC.2012.0526 . 23198999

